# Ferulic, Sinapic, 3,4-Dimethoxycinnamic Acid and Indomethacin Derivatives with Antioxidant, Anti-Inflammatory and Hypolipidemic Functionality

**DOI:** 10.3390/antiox12071436

**Published:** 2023-07-17

**Authors:** Panagiotis Theodosis-Nobelos, Georgios Papagiouvannis, Eleni A. Rekka

**Affiliations:** 1 Department of Pharmacy, School of Health Sciences, Frederick University, Nicosia 1036, Cyprus; hsc.pag@frederick.ac.cy; 2 Laboratory of Pharmaceutical Chemistry, School of Pharmacy, Aristotelian University of Thessaloniki, 54124 Thessaloniki, Greece; rekka@pharm.auth.gr

**Keywords:** ferulic acid, sinapic acid, cinnamic acid derivatives, inflammation, dyslipidemia, antioxidant activity, anti-inflammatory

## Abstract

A series of thiomorpholine and cinnamyl alcohol derivatives, conjugated with cinnamic acid-containing moieties, such as ferulic acid, sinapic acid and 3,4-dimethoxycinnamic acid, were synthesized and tested for their antioxidant, anti-inflammatory and hypolipidemic properties. An indomethacin ester with 2,6-di-*tert*-butyl-4-(hydroxymethyl)phenol was also prepared for reasons of comparison. The majority of the compounds demonstrated considerable antioxidant capacity and radical scavenging activity, reaching up to levels similar to the well-known antioxidant trolox. Some of them had an increased anti-inflammatory effect on the reduction of carrageenan-induced rat paw edema (range 17–72% at 150 μmol/kg), having comparable activity to the NSAIDs (non-steroidal anti-inflammatory drugs) used as reference. They had moderate activity in soybean lipoxygenase inhibition. All the tested compounds exhibited a significant decrease in lipidemic indices in Triton-induced hyperlipidemia in rats, whilst the most active triglycerides and total cholesterol decreased by 72.5% and 76%, respectively, at 150 μmol/kg (i.p.), slightly better than that of simvastatin, a well-known hypocholesterolemic drug, but with negligible triglyceride-lowering effect. Since our designed compounds seem to exhibit multiple pharmacological activities, they may be of use in occasions involving inflammation, oxidative stress, lipidemic deregulation and degenerative conditions.

## 1. Introduction

Since the definition of oxidative stress (OS) has evolved as the imbalance between the pro-oxidant and antioxidant mechanisms, in favor of the first, it became clear that such conditions may affect the redox circuitry and cellular integrity, leading to the formation of reactive oxygen and nitrogen species (ROS and RNS), together with lipid peroxidation products and oxidized protein adducts [1]. ROS formation stimulates the expression of redox-sensitive pro-inflammatory cytokines and caspases, which collaborate for the progression of diseases by the parallel activation of OS and inflammation and the mediation of the expression of anti-apoptotic and immune regulators that account for degenerative disorders and malignant conditions [2,3]. Cytokines, like interferon-γ, are key modulators for the expression of M1 macrophages, activating other pro-inflammatory genes (e.g., C-reactive protein) in an auto-inflammatory manner, giving rise to OS, in endothelial cells, in a bidirectional way [4].

Oxidized lipids induce the expression of IL-1β (Interleukin-1β), reduce paraoxonase-1 antioxidant activity and increase lipid hydroperoxides, in lipoproteins, and the activity of myeloperoxidase, with the parallel formation of malondialdehyde and covalent crosslinkages of apolipoproteins, deteriorating the oxidative and inflammatory components in the bloodstream [5,6]. Furthermore, hypercholesterolemia has been connected with low activity of antioxidant enzymes in the heart and reduced NO (nitric oxide) levels due to decreased transcription of constitutive cardiac nitrogen monoxide synthase, whilst the inducible form (iNOS) was unaffected in cholesterol-fed mice [7], aggravating the progression of contractile dysfunction, protein oxidation [8] and ischemia/reperfusion injury [9].

Anti-inflammatory compounds like non-steroidal anti-inflammatory drugs (NSAIDs) are widely applicable for various inflammatory conditions and have been shown to mediate anti-inflammatory and antioxidant paths in a direct and non-direct manner [10,11]. Nevertheless, their cyclooxygenase (COX) 1 and 2 inhibitory activity may adversely affect the gastric and duodenal function and the cardiovascular system due to a drastic decrease in the protective prostaglandins or the induction of leukotriene pathway with lipoxygenases (LOX) [12,13].

Cinnamic acid and its derivatives possess various properties covering the array of cellular protection and decrement of the factors of metabolic syndrome, with anti-inflammatory and anti-proliferative properties [14,15], whilst its derivative, ferulic acid, has shown similar activities together with direct antioxidant capacity, due to its phenolic moiety [16,17]. Sinapic acid is another derivative of the category of cinnamic with various disease-modifying activities, especially those implicated with oxidative stress [18,19]. Likewise, cinnamyl alcohol, due to the phenyl-acryl moiety, offers anti-inflammatory, lipid-lowering activity and lipoxygenase inhibitory activity to the acids that have been esterified with it [20]. Thiomorpholine is a scaffold with multifaceted actions in the cardiovascular and nervous system and increased hypolipidemic properties [21,22,23].

**Figure 1:**
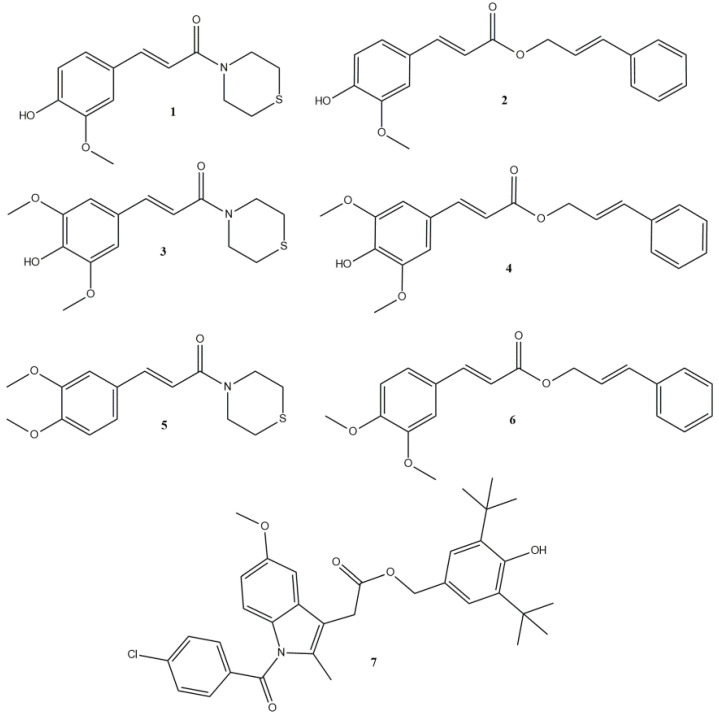
Structures of the synthesized compounds (compound **1**: (*E*)-3-(4-hydroxy-3-methoxyphenyl)-1-thiomorpholinoprop-2-en-1-one; compound **2**: (*E*)-cinnamyl 3-(4-hydroxy-3-methoxyphenyl)acrylate; compound **3**: (*E*)-3-(4-hydroxy-3,5-dimethoxyphenyl)-1-thiomorpholinoprop-2-en-1-one; compound **4**: (*E*)-cinnamyl 3-(4-hydroxy-3,5-dimethoxyphenyl)acrylate; compound **5**: (*E*)-3-(3,4-dimethoxyphenyl)-1-thiomorpholinoprop-2-en-1-one; compound **6**: (*E*)-cinnamyl 3-(3,4-dimethoxyphenyl)acrylate; compound **7**: 3,5-di-tert-butyl-4-hydroxybenzyl 2-(1-(4-chlorobenzoyl)-5-methoxy-2-methyl-1H-indol-3-yl)acetate).

**Figure 2:**
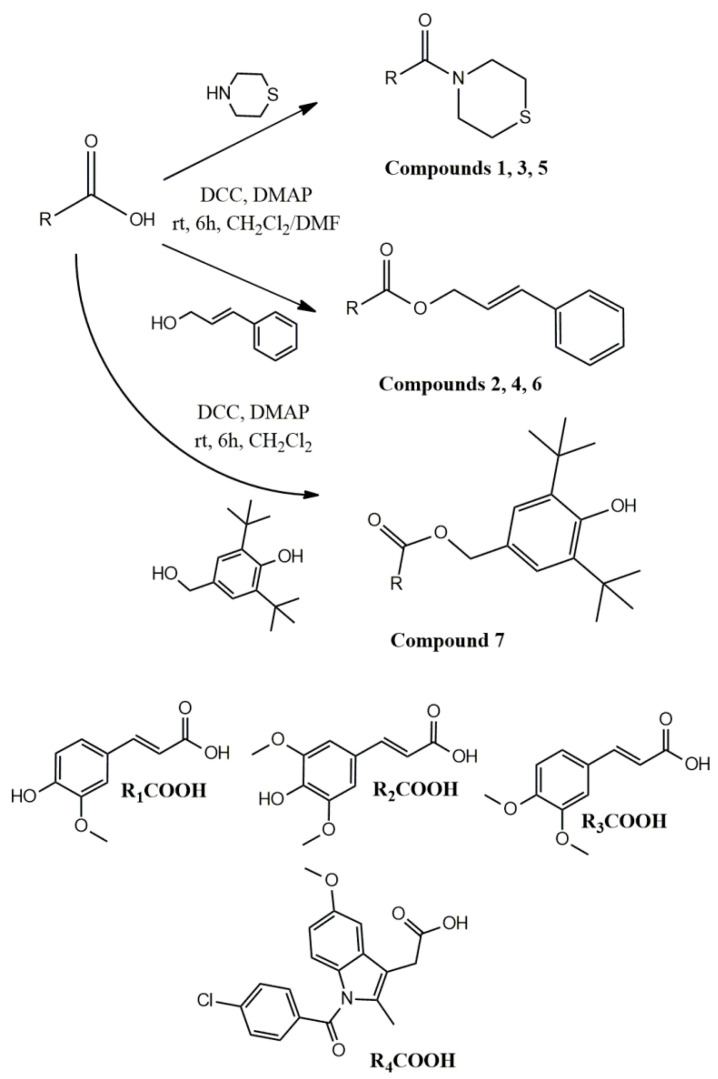
Synthesis of compounds **1**–**7**.

In view of all the above, in this research, we synthesized a series of amides with thiomorpholine (compounds **1**, **3**, **5**) and esters with cinnamyl alcohol (compounds **2**, **4**, **6**) (Figure 1) using cinnamic acid-bearing moieties, ferulic acid (R_1_-COOH), sinapic acid (R_2_-COOH) and 3,4-dimethoxy-cinnamic acid (R_3_-COOH) (Figure 2). An indomethacin with 2,6-di-*tert*-butyl-4-(hydroxymethyl)phenol hybrid (compound **7**) was also synthesized as a molecule that could combine both the anti-inflammatory characteristics of an NSAID and the molecular characteristics of butylated hydroxytoluene (BHT), a broadly used antioxidant that possess multiple activities and a low toxicity profile [24] (Figure 2). The antioxidant activity of the synthesized compounds, expressed as inhibition of rat hepatic microsomal membrane lipid peroxidation, as well as interaction with the stable radical 2,2-diphenyl-1-picrylhydrazyl (DPPH), was evaluated. Their effect on carrageenan-induced rat paw edema, in vitro activity on soybean lipoxygenase and hypolipidemic activity in vivo were also evaluated. The aim of this work is to investigate whether these hybrid compounds can lead to multifunctional derivatives that are able to restore OS, inflammatory conditions and lipid deregulation.

## 2. Materials and Methods

### 2.1. General

All commercially available chemicals of the appropriate purity were purchased from Merck (Kenilworth, NJ, USA) or Sigma (St. Louis, MO, USA). The IR spectra were recorded on a Perkin Elmer Spectrum BX FT-IR spectrometer (Waltham, MA, USA). The ^1^H NMR and ^13^C NMR spectra were recorded using an AGILENT DD2-500 MHz (Santa Clara, CA, USA) spectrometer. All chemical shifts are reported in δ (ppm), and signals are given as follows: s, singlet; d, doublet; t, triplet; m, multiplet. Melting points (mp) were determined with a MEL-TEMPII (Laboratory Devices, Sigma-Aldrich, Milwaukee, WI, USA) apparatus. The microanalyses were performed on a Perkin-Elmer 2400 CHN elemental analyzer (Waltham, MA, USA). Thin-layer chromatography (TLC silica gel 60 F254 aluminum sheets, Merck (Kenilworth, NJ, USA) was used to follow the reactions and the spots were visualized under UV light.

κ-Carrageenan and lipoxygenase type I-B from soybean were purchased from Sigma (St. Louis, MO, USA). For the in vivo experiments, Wistar rats (160–220 g, 3–4 months old) were kept in the Centre of the School of Veterinary Medicine (EL54 BIO42), Aristotelian University of Thessaloniki, University Campus, which is registered by the official state veterinary authorities (presidential degree 56/2013, in harmonization with the European Directive 2010/63/EEC). The experimental protocols were approved by the Animal Ethics Committee of the Prefecture of Central Macedonia (no. 270079/2500). 

### 2.2. Synthesis

#### General Method for the Synthesis of the Compounds **1**–**7**

In a solution of the corresponding acid (1 mmol), in dry CH_2_Cl_2_ with DMF in a 50/1 ratio, 4-dimethylamino-pyridine (0.2 mmol) and thiomorpholine (1.2 mmol), or the relevant alcohol (cinnamyl alcohol or 2,6-di-tert-butyl-4-(hydroxymethyl)phenol, 1.2 mmol), were added. After 15 min, N,N’-dicyclohexylcarbodiimide (DCC, 1.2 mmol) was added, and the mixture was stirred under nitrogen atmosphere overnight. The resulting mixture was filtrated and washed with solutions of 5% HCl, 5% NaHCO_3_ and saturated NaCl, respectively, and dried over Na_2_SO_4_. The resulting mixture was further purified with flash chromatography using petroleum ether and ethyl acetate as eluents.

*(E)-3-(4-hydroxy-3-methoxyphenyl)-1-thiomorpholinoprop-2-en-1-one* (Compound **1**): Flash chromatography (petroleum ether/ethyl acetate 2:1 and subsequently 1:1). White solid, yield 87%, m.p. 131–133 °C. ^1^H-NMR (CDCl_3_): δ 2.69–2.65 (m, 4H, -C**H**_2_-S-C**H**_2_-), 3.88–3.86, 3.97–3.93 (m, 4H, -C**H**_2_-N-C**H**_2_-), 3.92 (s, 3H, -OC**H**_3_), 5.92 (s, 1H, -O**H**), 6.67 (d, J: 15.3 Hz, 1H, Ar-CH=C**H**-), 6.91 (d, J: 8.2 Hz, 1H, aromatic ring-5), 6.98 (d, J: 1.8 Hz, 1H, aromatic ring-2), 7.09 (dd, J: 8.2 Hz, 1.8 Hz, aromatic ring-6), 7.60 (d, J: 15.3 Hz, 1H, Ar-C**H**=CH-). ^13^C-NMR (CDCl_3_): δ 25.54 (2C, -**C**-S-**C**-), 33.80 (2C, -**C**-N-**C**-), 55.99 (1C, -O**C**H_3_), 109.92 (1C, aromatic ring-2), 114.24 (1C, aromatic ring-5), 114.77 (1C, Ar-C=**C**-), 121.90 (1C, aromatic ring-6), 127.63 (1C, aromatic ring-1), 143.44 (1C Ar-**C**=C), 146.71, (1C, aromatic ring-3), 147.47 (1C, aromatic ring-4), 165.91 (1C, -**C**=O). Anal. Calcd for C_14_H_17_ΝO_3_S: C, 60.19; H, 6.13; Ν, 5.01. Found: C, 60.25; H, 6.31; Ν, 4.78.

*(E)-cinnamyl 3-(4-hydroxy-3-methoxyphenyl)acrylate* (Compound **2**): Flash chromatography (petroleum ether/ethyl acetate 4:1 and subsequently 2:1). White solid, yield 75%, m.p. 31–33 °C. ^1^H-NMR (DMSO-*d*_6_): δ 3.79 (s, 3H, -OC**H**_3_), 4.93 (d, J: 6.2 Hz, 2H, -O-C**H**_2_-), 6.40 (dt, J: 16.0, 6.2 Hz, 1H, Ar-CH=C**H**-CH_2_-O-), 6.64 (d, J: 15.9 Hz, 1H, Ar-CH=C**H**-C(O)-), 6.72 (d, J: 16.0 Hz, 1H, Ar-C**H**=CH-CH_2_-O), 6.96 (d, J: 8.3 Hz, 1H, ferulic ring-5), 7.39–7.21 (m, 5H, ferulic ring-2,6 cinnamic ring-3,4,5), 7.46 (d, J: 7.4 Hz, 2H, cinnamic ring-2,6), 7.61 (d, J: 15.9 Hz, 1H, Ar-C**H**=CH-C(O)-). ^13^C-NMR (DMSO-*d*_6_): δ 56.03 (1C, -O**C**H_3_), 64.79 (1C, -O-**C**H_2_-), 110.74 (1C, ferulic ring-2), 111.95 (1C, ferulic ring-5), 115.96 (1C, Ar-C=**C**-C(O)-), 123.28 (1C, ferulic ring-6), 124.44 (1C, Ar-C=**C**-CH_2_-O-), 126.94 (2C, cinnamic ring-2,6), 127.86 (1C, cinnamic ring-4), 128.49 (1C, ferulic ring-1), 129.04 (2C, cinnamic ring-3,5), 133.50 (1C, Ar-**C**=C-CH_2_-O-), 136.47 (1C, cinnamic ring-1), 145.41 (1C, Ar-**C**=C-C(O)-), 149.46 (1C, ferulic ring-3), 151.83 (1C, ferulic ring-4), 168.07 (1C, -**C**=O). Anal. Calcd for C_19_H_18_O_4_ × 0.7H_2_O: C, 70.66; H, 6.05. Found: C, 70.66; H, 6.12.

*(E)-3-(4-hydroxy-3,5-dimethoxyphenyl)-1-thiomorpholinoprop-2-en-1-one* (Compound **3**): Flash chromatography (petroleum ether/ethyl acetate 2:1 and subsequently 1:1). Viscous transparent oil, yield 79%. ^1^H-NMR (CDCl_3_): δ 2.69–2.65 (m, 4H, -C**H**_2_-S-C**H**_2_-), 3.92 (s, 6H, -OC**H**_3_), 4.02–3.93 (m, 4H, -C**H**_2_-N-C**H**_2_-), 5.76 (s, 1H, -O**H**), 6.66 (d, J: 15.3 Hz, 1H, Ar-CH=C**H**-), 6.74 (s, 2H, aromatic ring-2,6), 7.58 (d, J: 15.3 Hz, 1H, Ar-C**H**=CH-). ^13^C-NMR (CDCl_3_): δ 25.58 (2C -**C**-S-**C**-), 33.88 (2C, -**C**-N-**C**-), 56.39 (2C, -O**C**H_3_), 104.85 (2C, aromatic ring-2,6), 114.57 (1C, Ar-C=**C**-), 126.60 (1C, aromatic ring-1), 136.68 (1C, aromatic ring-4), 143.64 (1C, Ar-**C=**C-), 147.18 (2C, aromatic ring-3,5), 165.75, (1C, -**C**=O). Anal. Calcd for C_14_H_17_ΝO_3_S: C, 58.23; H, 6.19; Ν, 4.53. Found: C, 58.29; H, 6.07; Ν, 4.24.

*(E)-cinnamyl 3-(4-hydroxy-3,5-dimethoxyphenyl)acrylate* (Compound **4**): Flash chromatography (petroleum ether/ethyl acetate 4:1 and subsequently 2:1). Viscous transparent oil, yield 46%. ^1^H-NMR (DMSO-*d*_6_): δ 3.89 (s, 6H, -OC**H**_3_), 4,91 (d, J: 6.3Hz, 2H, -OC**H**_2_-), 6.37 (dt, J: 15.8 Hz, 6.3 Hz, 1H, Ar-CH=C**H**-CH_2_-O-), 6.63 (d, J: 15.3 Hz, 1H, Ar-CH=C**H**-C(O)-), 6.70 (d, J: 15.8 Hz, 1H, Ar-C**H**=CH-CH_2_-O-), 6.79 (s, 2H, sinapic ring-2,6), 7.05–7.24 (m, 5H, cinnamic ring), 7.62 (d, J: 15.3 Hz, 1H, Ar-C**H**=CH-C(O)-). ^13^C-NMR (DMSO-*d*_6_): δ 56.77 (2C, -O**C**H_3_), 64.32 (1C, -O-**C**H_2_-), 105.81 (2C, sinapic ring-2,6), 115.36 (1C, Ar-C=**C**-C(O)-) 120.89 (1C, Ar-C=**C**-CH_2_-O-), 127.01 (2C, cinnamic ring-2,6), 127.93 (1C sinapic ring-1), 128.14 (1C, cinnamic ring-4), 129.44 (2C, cinnamic ring-3,5), 136.01 (1C, Ar-**C**=C-CH_2_-O-), 137.67 (1C, cinnamic ring-1), 139.00 (1C, sinapic ring-4), 146.02 (1C, Ar-**C**=C-C(O)-), 148.04 (2C, sinapic ring-3,5), 169.73 (1C, -**C**=O). Anal. Calcd for C_20_H_20_O_5_: C, 70.57; H, 5.92. Found: C, 70.20; H, 6.18.

*(E)-3-(3,4-dimethoxyphenyl)-1-thiomorpholinoprop-2-en-1-one* (Compound **5**): Flash chromatography (petroleum ether/ethyl acetate 3:1 and subsequently 1:1). Viscous transparent oil, yield 77%. ^1^H-NMR (CDCl_3_): δ 2.67 (s, 4H, -C**H**_2_-S-C**H**_2_-), 3.90 (s, 3H, -OC**H**_3_), 3.91 (s, 3H, -OC**H**_3_), 4.05–3.92 (m, 4H, -C**H**_2_-N-C**H**_2_-), 6.68 (d, J: 15.3 Hz, 1H, Ar-CH=C**H**-), 6.85, (d, J: 8.2 Hz, 1H, aromatic ring-5), 7.01 (s, 1H, aromatic ring-2), 7.10 (d, J: 8.3 Hz, 1H, aromatic ring-6), 7.61 (d, J: 15.3 Hz, 1H, Ar-C**H**=CH-). ^13^C-NMR (CDCl_3_): δ 25.68 (2C, -**C**-S-**C**-), 33.86 (2C, -**C**-N-**C**-), 55.97 (1C, -O**C**H_3_), 56.02 (1C, -O**C**H_3_), 110.06 (1C, aromatic ring-2), 115.17 (1C, aromatic ring-5), 115.96 (1C, Ar-C=**C**-), 122.05 (1C, aromatic ring-6), 131.10 (1C, aromatic ring-1), 143.98 (1C Ar-**C**=C), 149.17 (1C, aromatic ring-3), 147.31 (1C, aromatic ring-4), 166.02 (1C, -**C**=O). Anal. Calcd for C_15_H_19_ΝO_3_S: C, 61.41; H, 6.53; Ν, 4.77. Found: C, 61.04; H, 6.17; Ν, 5.12.

*(E)-cinnamyl 3-(3,4-dimethoxyphenyl)acrylate* (Compound **6**): Flash chromatography (petroleum ether/ethyl acetate 4:1 and subsequently 2:1). Viscous transparent oil, yield 64%. ^1^H-NMR (DMSO-*d*_6_): δ 3.77 (s, 3H, -OC**H**_3_), 3.78 (s, 3H, -OC**H**_3_), 4.81 (d, J: 6.1 Hz, 2H, -O-C**H**_2_-), 6.42 (dt, J: 16.0, 6.1 Hz, 1H, Ar-CH=C**H**-CH_2_-O-), 6.60 (d, J: 15.9 Hz, 1H, Ar-CH=C**H**-C(O)-), 6.72 (d, J: 16.0 Hz, 1H, Ar-C**H**=CH-CH_2_-O-), 6.96 (d, J: 8.3 Hz, 1H, ferulic ring-5), 7.29–7.21 (m, 2H, ferulic ring-2, cinnamic ring-4), 7.36–7.31 (m, 3H, ferulic ring-6, cinnamic ring-3,5), 7.46 (d, J: 7.4 Hz, 2H, cinnamic ring-2,6), 7.62 (d, J: 15.9Hz, 1H, Ar-C**H**=CH-C(O)-). ^13^C-NMR (DMSO-*d*_6_): δ 56.00 (1C, -O**C**H_3_), 56.05 (1C, -O**C**H_3_), 64.73 (1C, -O-**C**H_2_-), 110.86, (1C, ferulic ring-2), 111.95 (1C, ferulic ring-5), 115.79 (1C, Ar-C=**C**-C(O)-), 123.45 (1C, ferulic ring-6), 124.41 (1C, Ar-C=**C**-CH_2_-O-), 126.92 (2C, cinnamic ring-2,6), 127.78 (1C, cinnamic ring-4), 128.43 (1C, ferulic ring-1), 129.12 (2C, cinnamic ring-3,5), 133.56 (1C, Ar-**C**=C-CH_2_-O-), 136.43 (1C, cinnamic ring-1), 145.41 (1C, Ar-**C**=C-C(O)-), 149.41 (1C, ferulic ring-3), 151.47 (1C, ferulic ring-4), 166.73 (1C, -**C**=O). Anal. Calcd for C_20_H_20_O_4_: C, 74.06; H, 6.21. Found: C, 73.98; H, 5.90.

*3,5-di-tert-butyl-4-hydroxybenzyl 2-(1-(4-chlorobenzoyl)-5-methoxy-2-methyl-1H-indol-3-yl)acetate* (Compound **7**): Flash chromatography (petroleum ether/ethyl acetate 10:1 and subsequently 2:1). Pale yellow solid, yield 67%, m.p. 61–63 °C. IR (Nujol) λ_max_: 3584 (O-H), 3339 (N-H), 1734 (C=O ester), 1684 (C=O amide), 1592, (C-C aromatic) cm^−1^. ^1^H NMR (CDCl_3_), δ (ppm): 1.43 (s, 18H, di-tert-butylated-CH_3_), 2.42 (s, 3H, C**H_3_**-C-N-), 3.73 (s, 5H, -C**H_2_**-C=O and -O-C**H_3_**), 5.08 (s, 2H, -O-C**H_2_**-Ar), 5.32 (s, 1H, aromatic -OH), 6.68 (dd, 1H, *J* = 9.0, 2.5 Hz, aromatic indole C6 H), 6.89 (d, 1H, *J* = 9.0 Hz, aromatic indole C7 H), 6.95 (d, 1H, *J* = 2.5 Hz, aromatic indole C4 H), 7.14 (s, 2H, aromatic di-tert-phenol C2, C5 H), 7.49 (d, 2H, *J* = 8.2 Hz, aromatic chloro-benzoyl C3, C5 H), 7.68 (d, 2H, *J* = 8.2 Hz, aromatic chloro-benzoyl C2, C6 H). ^13^C NMR (CDCl_3_) *δ*: 13.40 (1C, N-C-**C**H_3_), 30.15 (6C, C(**C**H_3_)**_3_**), 30.54 (1C, **C**H_2_-C=O), 35.31 (2C, **C**(CH_3_)_3_), 55.49 (1C, -O**C**H_3_), 67.66 (1C, Ar-**C**H_2_-O-), 101.30 (1C, indole C4), 111.72 (1C, indole C6), 112.69 (1C, indole C3), 114.86 (1C, indole C7), 125.65 (2C, phenyl **C2**, **C6**), 126.30 (1C, phenyl **C1**), 129.07 (2C, chlorobenzoyl C2, C6), 130.63 (1C, indole C3a), 130.76 (1C, indole C1a), 131.15 (2C, chlorobenzoyl C3, C5), 133.92 (1C, chlorobenzoyl C1), 135.85 (1C, chlorobenzoyl C4), 136.01 (2C, phenyl **C3**, **C5**), 139.20 (1C, indole C2), 153.96 (1C, phenyl **C4**), 155.96 (1C, indole C5), 168.18 (1C, CH_2_**C**=O), 170.80 (1C, N-**C**=O). Anal. Calcd for C_34_H_38_ClΝO_5_: C, 70.88; H, 6.65; Ν, 2.43. Found: C, 70.89; H, 6.29; Ν, 2.74.

### 2.3. In Vitro Biological Experiments

#### 2.3.1. In Vitro Lipid Peroxidation

Hepatic microsomal fraction from untreated rats was prepared. The incubation mixture contained heat-inactivated (at 90 °C for 90 s) microsomal fraction, corresponding to 2.5 mg protein/mL (final concentration) or 4 mM fatty acid residues, ascorbic acid (0.2 mM) in Tris–HCl/KCl buffer (50/150 mM), and the test compounds dissolved in dimethylsulfoxide (dimethylsulfoxide alone was used for control groups). The peroxidation reaction was initiated by the addition of a freshly prepared FeSO_4_ solution (10 μM), and the mixture was incubated at 37 °C. Aliquots (0.3 mL) were taken at various time intervals for 45 min. Lipid peroxidation was assessed spectrophotometrically (535 against 600 nm) by the determination of 2-thiobarbituric acid reactive material. All compounds, as well as dimethylsulfoxide, were tested and found not to interfere with the assay [25].

#### 2.3.2. In Vitro Scavenging of the Stable Radical 1,1-Diphenyl-2-picrylhydrazyl (DPPH)

Compounds (in absolute ethanol, final concentrations 50–200 μM) were added to an equal volume of ethanolic solution of DPPH (final concentration 200 μM) at room temperature (22 ± 2 °C). Ethanol without the addition of compound was used in case of control. Absorbance (517 nm) was recorded every 5 min for 30 min in total [25].

#### 2.3.3. In Vitro Evaluation of Lipoxygenase Activity

The reaction mixture (total volume 3 mL) contained 100 μL of the test compounds (100 μM) dissolved in absolute ethanol or the solvent (control), 200 μL of soybean LOX (250 u/mL) dissolved in 0.9% NaCl solution, and 2.6 mL Tris–HCl buffer, pH 9.0. The reaction was initiated by the addition of 100 μL sodium linoleate (100 μM) in the sample mixture (an equal volume of buffer being added to the reference solution). The reaction was followed for 7 min at 28 °C, recording the absorbance (234 nm) of a conjugated diene structure due to the formation of 13-hydroperoxy-linoleic acid. The performance of the assay was verified using NDGA as a reference. For the estimation of the type of inhibition, the described experiments were repeated, using sodium linoleate at a concentration (1 mM) higher than the saturating substrate concentration [26].

### 2.4. In Vivo Biological Experiments

#### 2.4.1. Carrageenan-Induced Paw Edema

A total of 0.1 mL of an aqueous solution of carrageenan (1% *w*/*v*) was injected i.d. into the right hind paw of rats, with the left paw serving as control. The tested compounds (suspended in water with a few drops of Tween 80) were given i.p. (0.15 mmol/kg) 5 min before the carrageenan administration, whilst water, instead of compound suspension, was administered in case of control. After 3.5 h, the hind paws were excised and weighed separately. The produced edema was estimated as paw weight increase [26].

#### 2.4.2. Effect on Plasma Cholesterol and Triglyceride Levels

A solution of Triton WR 1339 (tyloxapol) in saline was administered i.p. (200 mg/kg) to male rats once, and half hour later, the examined compound (0.15 mmol/kg) (suspended in water with a few drops of Tween 80) was given i.p. once. Control groups were treated with saline only instead of suspension of the compound. After 24 h, blood was taken from the aorta under hexobarbital anesthesia and used for the determination of plasma total cholesterol (TC) and triglyceride (TG) concentrations, using commercial kits, against standard solutions [26].

## 3. Results and Discussion

### 3.1. Synthesis

All the compounds were synthesized by direct amidation or esterification of the carboxylic group of the respective acids with thiomorpholine, cinnamyl alcohol or 2,6-di-tert-butyl-4-(hydroxymethyl)phenol, using *N*,*N*′-dicyclohexyl-carbodiimide (DCC), at room temperature and with high yields, in a range 46–87% (Figure 2). It is shown that the cinnamyl alcohol derivatives (compounds **2**, **4**, **6**) gave lower yields than that of thiomorpholine, perhaps due to stereochemical reasons and difficulty in the purification of the resulted compounds, with flash column chromatography, from the unreacted cinnamyl alcohol. Other methods of purification of these compounds, like recrystallization, were not efficiently applicable since the compounds were either oils or low melting point solids.

### 3.2. Biological Evaluation

#### 3.2.1. Effect on Lipid Peroxidation

The effect of the synthesized compounds on rat hepatic microsomal membrane lipid peroxidation, expressed as IC_50_ values after 45 min of incubation, is shown in Table 1. Trolox was included for comparison.

Compounds **5** and **6** were inactive in this experiment, and this may derive from the absence of an easily abstracted hydrogen atom from their structure. As for the rest of the compounds, they showed considerable activity, reaching up to 70% of that of trolox. This could be explained by the presence of the extended conjugation of the phenyl-acrylic moiety and the phenolic hydroxyl group that offer lipid peroxidation inhibitory ability. Towards this direction, the relatively increased lipophilicity of the compounds may also assist (Table 2), making the access to the lipid phase more facile.

The results of the ferulic acid compounds **1** and **2** are in accordance with previous findings from our laboratory [27]. Nevertheless, the activity of compound **2** is much higher, and a potential explanation may be the existence of two molecular parts with extended conjugation on the structure. However, it seems that this effect is not enough for compound **4**. Moreover, compound **4** was not easily soluble in the experiment’s medium, making it hard to express its full range of activity. We have previously shown that compounds bearing lipophilic moieties may result in low dissolution and reduced activity in this experiment that do not relate to their antioxidant potential [25]. Furthermore, the more increased polar area of compounds **3** and **4** may also negatively affect their accessibility to the lipid phase. Thiomorpholine structure has been shown to result in antioxidant activity, together with multifaceted properties [23], and the antioxidant potential has been seen in various experimental assays, including DPPH [28]. The presence of phenyl and biphenyl hybrid seems to be of additional effect in the lipid peroxidation inhibition and the antioxidant status in general, whilst the incorporation of electron donating groups like phenol or thiol seems to be another very important factor [29]. As for compound **7**, the presence of butylated-hydroxy phenyl moiety seems to give antioxidant capacity similar to that of the reference compound. This finding may further contribute to the reduction of the in vivo-induced lipid peroxidation of indomethacin [30], relieving the tissue damage that this NSAID may provoke.

**Figure 3:**
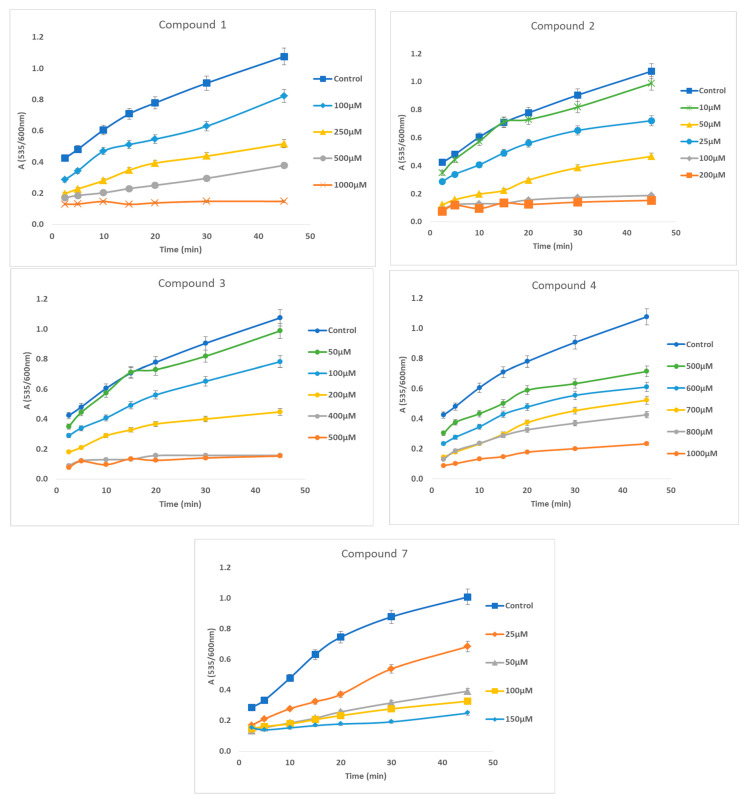
The effect of various concentrations of the active compounds **1**, **2**, **3**, **4** and **7** on the time course of lipid peroxidation. For control groups, dimethylsulfoxide alone, instead of solution of the tested compound in dimethylsulfoxide, was used. All determinations were performed in triplicate, and the standard deviation was always within ±10% of the mean value.

The time course of lipid peroxidation, as affected by various concentrations of the active derivatives, compounds **1**, **2**, **3**, **4** and **7**, is shown in Figure 3.

#### 3.2.2. Scavenging of DPPH

The antioxidant activity of our compounds was also evaluated by their ability to scavenge the stable, N-centered free radical 1,1-diphenyl-2-picrylhydrazyl (DPPH). The per cent interaction between the active compounds and DPPH is shown in Table 3.

As expected from the lipid peroxidation experiments, compounds **5** and **6** had no activity in this test, and surprisingly, compounds **2** and **4**, which were active at lipid peroxidation inhibition, did not show radical scavenger activity. This may derive from the dual *trans*-stereochemistry of the compounds, making the approach to the N-centered radical difficult, and not due to the hydrogen atom donating inability that may explain their activity on lipid peroxidation. Compounds **1**, **3** and **7** showed good radical interaction ability, in a concentration ratio of almost 2:1 with DPPH for compound **1**, and 1:1 for compounds **3** and **7**. Although results for compound **7** are in parallel with the previous experiment, compounds **1** and **3** show profound radical scavenging ability compared to lipid peroxidation inhibition, and this may partly derive from the homogenous reaction mixture of this experiment. These results are related to the phenolic moiety and the stabilization of the formed radical into the aromatic ring for such natural source-derived phenolic acids [31].

**Figure 4:**
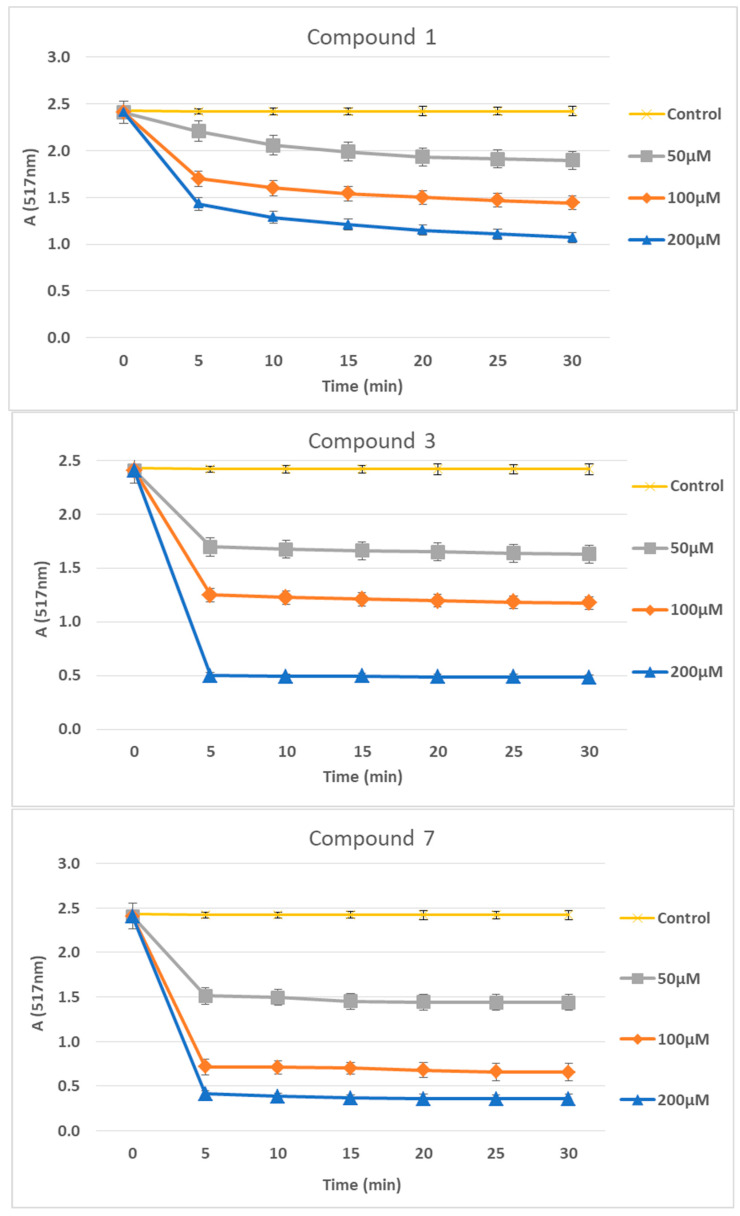
Time course of DPPH scavenging with the active compounds **1**, **3** and **7**. In the control group, ethanol alone was used in the place of compounds. All determinations were performed in triplicate, and standard deviation was always within ±10% of the mean value.

The time course of DPPH scavenging with the active compounds **1**, **3** and **7**, is shown in Figure 4.

#### 3.2.3. Effect on Acute Inflammation

Carrageenan-induced paw edema is a well-known biphasic experimental protocol for acute inflammation, where at the early phase, the release of histamine, serotonin and kinins takes place, with neutrophil infiltration and prostaglandin and cytokine release involved later (>2.5 h post-administration) [32].

The effect of the synthesized compounds on acute inflammation, applying the carrageenan paw edema model, as well as the anti-inflammatory activity of ibuprofen, tolfenamic acid and indomethacin, used as a reference, are shown in Table 4.

The compounds could reduce paw edema from about 17% up to 72% at 150 μmol/kg, i.p., 3.5 h after carrageenan administration. The most active compound **7** was the most potent antioxidant, indicating a possible implication of antioxidant potential of the examined compounds in acute inflammation. Thiomorpholine derivatives were more active compared to those of cinnamyl alcohol for all the tested cinnamic derivatives. Their activity was greater than some well-established NSAIDs, such as ibuprofen and tolfenamic acid, or even the more potent indomethacin that was 21% and 42% less drastic than compound **1** and **7**, respectively. Cinnamic acid and its derivatives have been found to decrease carrageenan-induced inflammation and suppress iNOS, COX-2 and NF-κB expression, as well as TNF-α [33,34]. Furthermore, we have shown that these antioxidant moieties seem to enhance the anti-inflammatory potency in vivo [35], whilst butylated hydroxytoluene derivatives may give highly active anti-inflammatory hybrids when combined with NSAIDs, resulting in dually acting derivatives [36]. Ferulic acid has been shown to offer anti-inflammatory activity in vivo, reversing the cerebral ischemia in rats and the infarct size, acting as a dual antioxidant and inflammation modulatory molecule [37], whilst sinapic acid has also been linked with lipid peroxidation decrease in vivo and parallel improvement of inflammatory conditions histologically and on inflammatory markers improvement [38,39]. Additionally, thiomorpholine insertion has been shown to improve the pleiotropic activity of antioxidant derivatives [25], and these clues may partly explain the anti-inflammatory action of our compounds.

#### 3.2.4. Effect on Lipoxygenase

Eicosanoids are synthesized from arachidonic acid, mainly via two pathways. The first implicate is COX enzymes, and the second is the production of leukotrienes. The latter is responsible for various inflammatory reactions like rheumatoid arthritis, cardiovascular and neurodegenerative events [40,41]. Additionally, LOX inhibition may assist in the decreased oxidation of lipids and foam cell formation [42]. The inhibition of soybean lipoxygenase by the synthesized compounds is shown in Table 5. Indomethacin and NDGA, a redox-type LOX inhibitor, are included for comparison.

Most of the compounds had mediocre activity. Compound **7**, with high antioxidant, radical scavenging and in vivo anti-inflammatory potential, showed the best activity, followed by compound **2**, which had good lipid peroxidation inhibitory activity but relatively moderate anti-inflammatory activity. Compounds **5** and **6** were active despite the complete absence of any direct antioxidant effect, as is the case with our previous findings with dimethoxy-cinnamic derivatives [43]. Thus, we can assume that our compounds may not affect the free radicals formed by LOX, rather the binding of the substrate to the active center of the enzyme, since their antioxidant effect does not seem closely related to their LOX inhibitory effect. Cinnamic acid derivatives have been shown to react with lipoxygenase, with the oxygen of the carbonyl, forming hydrogen bonds, whilst hydroxyl groups in the structure seem to offer more effective binding, leading to significant binding energy to the protein [44]. 4-methoxy and 3,4-dimethoxy cinnamic derivatives have been shown to be implicated in soybean lipoxygenase inhibition, whilst 3,4,5-trimethoxy substitution seems to be related to diminution in the inhibitory activity [45]. Lastly, it can be deduced that our compounds act in a competitive manner with linoleic acid since no inhibition could be detected using linoleic acid at 1 mM concentration (higher than the saturating substrate concentration) under the same experimental setting.

#### 3.2.5. Effect of the Synthesized Compounds on Hyperlipidemia

Lipid levels and systemic inflammation are interconnected, with a decrease in the first resulting in a decrease in the latter, together with a decrement in the risk of cardiovascular events and plaque formation [46,47]. Widely applied hypocholesterolemic statins seem to offer, additionally to lipid-lowering effects, reduction of pro-inflammatory cytokines and chemokines, with a decrease in ROS and oxidized LDL (low density lipoprotein) and endothelial-NOS upregulation, reducing macrophage infiltration at atheromatic level [48,49].

Our most active in vivo anti-inflammatory cinnamic acid derivatives were tested for their anti-dyslipidemic activity in Triton-induced hyperlipidemia in rats. The systemic administration of Triton-WR1339 in rats results in the elevation of plasma cholesterol, intermediate density lipoproteins, LDL cholesterol, and especially, triglyceride levels with maximal accumulation after 24h, mainly due to inhibition of lipoprotein lipase and induction of 3-hydroxy-3-methyl-glutaryl-CoA (HMG-CoA) reductase [50,51]. The administration of the compounds goes almost in parallel with the tyloxapol administration, and blood is collected after 24 h of administration [50,51]. Results of the effect of the tested compounds, together with simvastatin, on Triton WR1339 (tyloxapol)-induced hyperlipidemia are shown in Table 6.

All the tested compounds caused significant decreases in total cholesterol and triglycerides, reaching up to 76% and 72.5% for each marker, respectively. With the exception of compound **2**, all the compounds reduced both lipidemic indices in a similar manner, despite the sharp increase in triglycerides, due to the applied protocol. The high activity of compounds **1–3** may partly rely on their antioxidant activity and their in vivo and in vitro anti-inflammatory activity, and perhaps this is based on their whole molecular entity since cinnamyl alcohol alone bears low anti-inflammatory characteristics [20]. Thus, the multi-effective properties of the compounds may assist the improved management of the lipids, offering the ability to the organism to cope with them in a decreased inflammatory and pro-oxidant state. Our results in this experiment come up to a very solid argument that the thiomorpholine-containing structures gave better results in lipid decrease, in comparison to those containing cinnamyl alcohol. There was a decrease in lipids that was similar or better, in cases of compounds **1** and **5**, of that of simvastatin, whilst statins’ effect on triglycerides is negligible, in contrast to our compounds. The high effect of thiomorpholine moiety on lipids is in accordance with our previous studies [22,25], pointing out that this moiety may be an independently positive modulator of the lipidemic indices. The effect on lipids is in parallel with the anti-inflammatory effect in vivo, dictating the ambivalent effects the compounds may have towards both conditions.

## 4. Conclusions

In conclusion, with the preparation of the above-described compounds with thiomorpholine, cinnamyl alcohol and 2,6-di-tert-butyl-4-(hydroxymethyl)phenol, we aimed to yield structures with at least two or more potent activities (antioxidant, anti-inflammatory and hypolipidemic).

The thiomorpholine derivatives were of increased anti-inflammatory effect in vivo compared to those bearing the cinnamyl alcohol moiety. The incorporation of an antioxidant structure and an anti-inflammatory molecule seemed to offer increased inflammation regulatory potency in comparison to the anti-inflammatory effect alone, as is the case for compound **7**. However, the antioxidant effect was not a limiting factor for such an activity, as shown by the anti-inflammatory potential of compounds **5** and **6**. The phenolic structure seems to be an integral part of the antioxidant effect and reducing capacity of the compounds, and the extended conjugation or the electron-donating substituents seem to add on towards this effect, although other physicochemical factors like lipophilicity and the polar surface of the compounds are also related to this.

Since oxidative stress, inflammation and hyperlipidemia seem to be related in an ambivalent way, all the tested compounds for hyperlipidemia gave a substantial decrease in lipid markers, whilst they had, in a similar way, anti-inflammatory efficiency, and most of them also had antioxidant capacity. Thiomorpholine incorporation seems to offer a high hypolipidemic effect, with cinnamyl alcohol following in a very efficient manner. Thus, compounds **1**, **3** and **5** were the most active hypolipidemics and anti-inflammatories tested, with additional antioxidant effects for compounds **1** and **3**.

By applying a targeted structural alteration, pleiotropic derivatives could evolve, affecting the progression of multicausal diseases of immune, cardiovascular and CNS origins. This is the scope of this article, taking into account that multi-drug therapies may account for increased adverse effects, as well as pharmacokinetic and pharmacodynamic interactions. In case one molecule possesses several actions, efforts like this may assist towards the diminution of untoward effects after proper optimization of the compounds.

## Figures and Tables

**Table 1:** Effect of the synthesized compounds on rat microsomal membrane lipid peroxidation.

Compound	Inhibition of Lipid Peroxidation: IC_50_ (μΜ) ^#^
**1**	260
**2**	43
**3**	169
**4**	680
**5**	-
**6**	-
**7**	36
**Trolox**	25

^#^ After 45 min of incubation. Trolox: 6-hydroxy-2,5,7,8-tetramethylchroman-2-carboxylic acid; -: practically inactive or of very low potency. All determinations were performed in triplicate, and the standard deviation was always within ±10% of the mean value.

**Table 2:** Calculated physicochemical properties of the synthesized compounds: Molecular Weight MW), total polar surface area (TPSA), number of H donors and acceptors, lipophilicity (ClogP) and violations of “Lipinski’s rule of 5”.

Compound	MW	TPSA (Å^2^)	ClogP	H Acceptors	H Donors	Violations
**1**	279.35	49.77	1.68	4	1	0
**2**	310.34	55.76	4.02	4	1	0
**3**	309.38	59.00	1.46	5	1	0
**4**	340.37	64.99	3.80	5	1	0
**5**	293.38	38.77	2.16	4	0	0
**6**	324.37	44.76	4.50	4	0	0
**7**	576.12	76.07	9.14	6	1	2

TPSA and ClogP calculated with ChemBioDraw Ultra 12.0.

**Table 3:** Interaction of compounds **1**, **3**, **7** and **trolox** at various concentrations with DPPH (200 μΜ) ^a^.

Compound	Percent Scavenging of DPPH (200 μΜ)		
	200 μΜ	100 μΜ	50 μΜ
**1**	55.6	40.2	21.3
**3**	80.0	51.2	32.3
**7**	85.2	67.9	40.3
**Trolox**	92.0	90.0	38.0

^a^ After 30 min of incubation. Trolox: 6-hydroxy-2,5,7,8-tetramethylchroman-2-carboxylic acid. All determinations were performed in triplicate, and standard deviation was always within ±10% of the mean value. Compounds **2**, **4**, **5** and **6** were inactive in this experiment.

**Table 4:** Effect of compounds **1**–**7**, ibuprofen, tolfenamic acid and indomethacin on carrageenan-induced rat paw edema ^a^.

Compound	x% Edema Reduction
**1**	53.0 **
**2**	25.0 *
**3**	31.0 *
**4**	17.0 *
**5**	32.8 **
**6**	22.7 *
**7**	72.0 **
**Ibuprofen**	36.0 *
**Tolfenamic acid**	24.0 **
**Indomethacin**	42.0 **

^a^ The effect on edema is expressed as percent of inhibition of edema in comparison to controls. All compounds were administered i.p. at a dose of 0.15 mmol/kg of body weight. Each value represents the mean obtained from 5 to 6 animals. Significant difference from control: * *p* < 0.01, ** *p* < 0.001 (Student’s *t*-test).

**Table 5:** Inhibitory activity of compounds **1**–**7**, indomethacin and NDGA (concentration 100 μM) against soybean lipoxygenase ^a^.

Compound	% Inhibition
**1**	20
**2**	56
**3**	-
**4**	-
**5**	41
**6**	49
**7**	81
**Indomethacin**	-
**NDGA**	94

^a^ After 7 min of incubation. NDGA: nordihydroguaiaretic acid; -: inactive. All determinations were performed in triplicate, and the standard deviation was always within ±10% of the mean value.

**Table 6:** Effect of the synthesized compounds **1**, **2**, **3**, **5** and **6**, together with simvastatin, on Triton WR1339 (tyloxapol)-induced hyperlipidemia.

Compound	% Reduction		
	Dose (i.p.) (μmol/kg)	TC ^a^	TG ^b^
**1**	150	72.6 **	70.3 *
**2**	150	66.4 **	43.2 ***
**3**	150	68.7 ***	64.5 ***
**5**	150	76.0 ***	72.5 ***
**6**	150	46.3 ***	49.5 ***
**Simvastatin**	150	73.0 ***	-

^a^ TC: Total cholesterol; ^b^ TG: Triglycerides. Tyloxapol: 200 mg/kg, i.p. -: not statistically significant result. Significant difference from hyperlipidemic control group: * *p* < 0.03, ** *p* < 0.005, *** *p* < 0.001 (Student’s *t*-test).

## Data Availability

The data presented in this study are available on request from the corresponding author.
